# Potential Conflicts of Interest Arising from Dualism of Loyalty Imposed on Employees of Medical Institutions—Findings and Tools for Ethics Management

**DOI:** 10.3390/medicina59091598

**Published:** 2023-09-04

**Authors:** Rodica Gramma, Bianca Hanganu, Oleg Arnaut, Beatrice Gabriela Ioan

**Affiliations:** 1 Doctoral School, State University of Medicine and Pharmacy Nicolae Testemițanu, 2004 Chisinau, Moldova; rodica.gramma@gmail.com (R.G.); oleg.arnaut@usmf.md (O.A.); 2 3rd Medical Sciences Department, Grigore T. Popa University of Medicine and Pharmacy, 700115 Iasi, Romania; beatrice.ioan@umfiasi.ro

**Keywords:** dual loyalty, conflicts of interest, ethical management, tools, medical practice

## Abstract

*Background and Objective:* Doctors should have full loyalty to their patients, while patients should be able to trust that physicians will act only in their best interests. However, doctors may be faced with situations where they must choose between the patient’s interests and those of a third party. This article presents the results of a study that aimed to identify situations of duality in the decision-making process of medical workers, which can compromise their ethical behavior. *Materials and Methods:* A cross-sectional study was carried out on a sample of 1070 participants, employed in 120 healthcare facilities in the Republic of Moldova. An online questionnaire was completed anonymously. Descriptive statistics for discrete data were performed by estimating absolute and relative frequencies. To perform the multivariate analysis, the logistic regression was applied. *Results:* A large number (74.4%) of respondents admitted that they had faced situations of conflicts of interest. Every third respondent (35.3%) had experienced ethical dilemmas when access to expensive treatments should be ensured. Every fourth respondent experienced a conflict between the patient’s interests and those of the institution (26.1%) or the insurance company (23.3%). As age increases, the probability of reporting the dilemma decreases. Physicians reported such dilemmas almost 3 times more often than nurses. A low rate of staff sought support when faced with dilemmas. Half of the respondents (50.6%) preferred to discuss the problem only with a colleague, and 40.1% preferred to find solutions without anyone’s help. There were significant gaps within organizations in terms of the ethical dimension of the decision-making process. *Conclusions:* Managers should adopt clear institutional policies and tools to identify and prevent situations of dual loyalty. Ethical support should be offered to employees facing such situations. The need to promote an institutional climate based on trust and openness becomes evident.

## 1. Introduction

Since its inception, medical practice has been guided by ethical codes that emphasize loyalty to the patient and pursuing the patient’s best interests. Whether it is the Hippocratic Oath in its original version or the various updated versions [1], or the Geneva Declaration [2], doctors are required to pursue the best interests of the patient above all, providing medical services in “full technical and moral independence” [3]. Moreover, according to the WMA International Code of Medical Ethics, “a physician shall owe his patients complete loyalty and all the resources of his science” [4].

With this fundamental principle of medicine in mind, patients trust the decisions doctors make from their first meeting, based on the belief that the doctor will act in their best interests [5].

However, in addition to caring for patients, doctors have other obligations, which are not necessarily aimed at the patient’s interests or benefit. First of all, doctors are employees of an institution and are involved in contractual and subordinate relations with their superiors. Moreover, doctors are often faced with other competing interests that directly or indirectly influence the performance of the medical act. These interests include those of other patients, members of the patients’ families, colleagues, hospital administrators, and insurance companies. Thus, putting patients’ interests first sometimes becomes difficult for doctors [5].

Dual loyalty in medicine is a “clinical role conflict between professional duties to a patient and obligations, expressed or implied, to the interests of a third party, such as an employer, insurer, or the state” [6]. Thus, dual loyalty dilemmas occur when the health care professional’s role as healer conflicts with their obligations to a third party [7].

The problem of dual loyalty abounds in medicine, and the ethical dilemmas associated with dual loyalty can compromise the ethical behavior of physicians, causing them to participate, knowingly or unknowingly, in the human rights abuses committed by a third party [7].

One area that involves a high risk of dual loyalty is the work of doctors assisting people in state custody (prisoners) [6]. In this context, classic conflicts generated by dual loyalties include the participation of doctors in the force-feeding of hunger strikers, acts of torture, and infliction of the death penalty [7]. In the penitentiary environment, the doctor must distinguish between “the penitentiary doctor” and “the doctor in the penitentiary” in order to clearly highlight in whose interest they are acting. This area is particularly targeted for regulation by many international structures and organizations [8,9,10].

In order to clarify doctors’ actions with greater certainty, in cases where a violation of someone’s rights and freedoms is alleged, often there are special legal provisions that strictly regulate the doctor’s duty to report the case, thus violating the confidentiality of the patient’s medical information. For example, the legislation of the Republic of Moldova requires the mandatory reporting of gunshot wounds, cold weapon injuries, child abuse, and rape to the police. However, there are many instances where rape victims will ask doctors for assistance but do not wish the police to be informed. This is especially the case when the rape occurs in small a community or rural region where the population knows each other and the victim does not want to be blamed and stigmatized. Thus, the doctor ends up facing a dilemma between moral obligations and medical ethics and the provisions of the law.

However, in their daily work, health care professionals face many other situations of dual loyalty, arising from the influence exerted by various third parties on the doctor–patient relationship. Dual loyalty poses particular challenges for health care professionals when subordinating the patient’s interests to those of a third party risks violating the patient’s fundamental rights [7].

Thus, from an ethical standpoint, dual loyalty is loyalty to two separate interests that potentially entails a conflict of interest, i.e., when an individual is involved in two different interests that work against each other. That is, when acting in the personal or organizational interest negatively affects the decisions regarding a third party [11].

The perception of dual loyalty is related to the moral behavior and ethical values that guide an employee, while a conflict of interest signifies action and the implementation of decisions made following dual reflection, which often has legal consequences.

In the medical field, a conflict of interest occurs when a doctor or a medical institution, in carrying out their activity, violates the principles of professional or institutional ethics. In doing so, they may also incur legal action arising from pursuing their own interest, or those of a third party, to the detriment of the patient’s interests [12]. The third party in this case can be the patient’s family, the insurance company, drug companies, the government/state, or the doctor’s colleagues [2,7]. Conflicts of interest risk compromising the physicians’ loyalty to their patients and often even their clinical judgment [13].

Although a physician’s conflicts of interest do not always cause direct harm to the patient, they can trigger risk factors in this regard [14]. Conflicts of interest can influence the way in which doctors make their decisions and take actions, sometimes without being fully aware of a possible bias. A specific example refers to the relation doctors have with drug companies, when gifts or payments can indirectly influence the way in which doctors present the results of a study involving a specific drug. Moreover, those who also work in the academia, teaching future doctors, can be influenced by the relation they have with drug companies when teaching about specific drugs. Not least, the same gifts and payments offered by drug companies can influence the treatment doctors recommend to their patients. Likewise, when doctors are themselves involved in this type of business (e.g., owning testing facilities or treatment centers), they tend to refer their patients to these services for their own financial benefit [15].

Thus, the involvement of doctors in activities intended for their own post-graduate training; the education of medical students; or involvement in clinical trials aimed at the discovery of new drugs, treatments or medical devices represents favorable contexts for building relationships with third parties, such as pharmaceutical companies. These relationships fall outside the doctor–patient relationship, sometimes reaching the level of business relationships [15], generating personal profit, and even progressing to acts of corruption and informal payments [16]. At the same time, the conflict of values with regard to the doctor’s loyalty to the patient can extend much wider, conditioned by both medical and non-medical factors such as, for example, religious visions or beliefs.

The situations described above provide a strong emotional context for the doctors involved, who must make important decisions regarding their professional behavior in the light of dilemmas and conflicts of values. At the same time, decision making in the context of ethical dilemmas in professional activity is a complex process, involving several factors—the doctor’s own visions and beliefs, the circumstances of the work environment, the attitude of the administration, the organizational values and traditions of the medical institution where the doctor works, the doctor’s personal experiences accumulated during their career, etc. To manage ethics in a medical institution, it is important that doctors’ decisions taken in dilemma situations are correct, from the point of view of professional ethics, but also in accordance with the law.

Ethics management in institutions is a new discipline within the general management field, which aims to develop and apply tools and mechanisms for monitoring, preventing, and solving ethical problems and conflicts in the institution in order to increase the quality of services and the satisfaction of beneficiaries [17,18].

Thus, the managerial framework of a medical institution must implement tools and mechanisms to ensure that risk situations of dual loyalty are identified; support and advice are offered when a health care professional faces dual loyalty situations; and moral values are promoted in the decision-making process of employees [19].

A research project entitled “Ethics Management in Healthcare Facilities for Respecting Human Rights in the Provision of Health Services” was carried out in the period 2022–2023 in the Republic of Moldova. This multidimensional evaluation of ethics management in medical institutions focused on 3 distinct aspects: (1) the analysis of the ethical policies and mechanisms adopted by the institution; (2) the evaluation of the ethical behavior of the employees; and (3) the determination of the particular competences of the employees with regard to certain ethical issues. Among the investigated aspects, the phenomenon of dual loyalty was also included, in terms of both its presence in the decision-making process among employees and the institutional context that influences the final decision of the health care professional. Although the scope of the research carried out was much broader, in this article we will only present the aspects related to the phenomenon of dual loyalty and conflicts of interest that have been identified.

## 2. Materials and Methods

A cross-sectional study was carried out, which included a sample of 1070 participants (at the margin of error ±3% and the expected frequency at the level of 50%), employed in hospitals and primary care institutions in the Republic of Moldova. Employees of medical institutions in the Republic of Moldova (about 120 medical institutions) with at least one year of professional activity were invited to participate in the study.

Taking into account the sensitivity of the issues addressed and the intention to provide equal opportunities for all potential participants to take part in the study, but also to exclude the influence of the researcher and the medical institution’s administration on the quality of the answers provided, the questionnaire was transposed into a Google Forms format (https://forms.gle/qcdxU1Bduy6tDzRn9, accessed on 10 February 2023) and later distributed through the human resources departments of the medical institutions and professional associations, as well as through social networks.

The questionnaire contained 48 questions to identify problems related to ethical dilemmas faced by the employees of the institution, the ways and possibilities identified by the employees to solve the problems, the employees’ assessment of the organizational climate and the factors that determine it, the existing ethical tools in the institution, as well as the assessment of the conditions and level of respect for the patient’s rights in the process of providing medical services. The questionnaire consisted of both closed- and open-ended questions. For the closed-ended questions, a 5-point rating scale and single-choice or multiple-choice matrixes were applied. The respondents were also given the possibility to provide their own answers through the inclusion of an open answer option, “Other”. In order to measure employees’ attitudes or perceptions, some semantic differential questions were also applied (for example, the appreciation of the image of the institution or the image of the manager). At the same time, the questionnaire included a separate open question through which the respondents were asked to formulate proposals and suggestions for improving the management of ethics in the institution where they work.

When invited to take part in the study, the respondents were assured that their participation would be confidential and that no personal data would be recorded (the completion of the questionnaire was done anonymously). The study was approved by the Research Ethics Committee of the State University of Medicine and Pharmacy “Nicolae Testemițanu” (Decision no. 1 of 16 February 2022).

Statistical Analysis of Data

Descriptive statistics for discrete data were performed by determining absolute and relative frequencies, completed with 95% confidence intervals (95% CI). Because the dependent variables were dichotomous, to perform the multivariate analysis, logistic regression was applied with the estimation of the B coefficient, the Standard Error (S.E.), the Wald statistic, degrees of freedom (df), the odds ratio (OR, Exp(B), as well as the confidence interval for the odds ratio. The program used for the statistical analysis was IBM SPSS (Statistical Package for the Social Sciences) Statistics (version 26), IBM company (Endicott, NY, USA).

## 3. Results

### 3.1. Descriptive Analysis

Physicians constituted 64.5% (N = 690) of the total number of respondents, with the other participants being represented by nurses (31.8%, N = 340) and non-medical staff employed in medical institutions (3.7%, N = 40). The number of specialists in the medical specialties (24.4%, N = 261, 95% CI, 21.9, 27.0) was close to that of specialists in surgical specialties (27.7%, N = 296, 95% CI, 25.0, 30.4), followed by the group of family doctors (16.9%, N = 181, 95% CI, 14.8, 19.3), obstetricians and gynecologists (11.1%, N = 119, 95% CI, 9.3, 13.1), pediatricians (10%, N = 107, 95% CI, 8.3, 11.9), and other specialties in lower rates. The age for all categories of participants is given in Table 1.

Given that the physicians taking part in the study belonged to one of six possible disciplines (Family Medicine, Oncology, Pediatrics, Surgery, Therapy, or Gynecology) with no hierarchy, we considered this feature a nominal one. This, in turn, generated some problems in the conditions of the multivariate data analysis. As a solution, we performed one hot encoding procedure, transforming this column into another six, with each one representing a separate category with 0, 1 coding.

The analysis of the data showed that the participants in the study were frequently faced with situations of dual loyalty that led to conflicts of interest.

### 3.2. Analysis of Situations of Dual Loyalty and Conflicts of Interest Faced by Medical Professionals

#### 3.2.1. The Obligation to Promote the Interests of the Institution

Every fourth respondent (26.1%, N = 278 95% CI, 23.5, 28.8) recognized the existence of a conflict between the economic interests of the institution where they worked and the interests of the patient, confirming that the manager asked them to save institutional resources at the expense of the patient’s necessary treatment. At the same time, every third respondent (35.3%, N = 378) faced ethical dilemmas when they had to ensure fair patient access to complex diagnostic investigations and expensive treatments, with 17.1% (N = 183, 95% CI, 14.9, 19.4) facing such dilemmas very often and 18.2% (N = 195, 95% CI, 16.0, 20.6) facing such dilemmas sometimes.

Every tenth respondent (10.6%, N = 113, 95% CI, 8.8, 12.5) admitted that they personally found themselves in situations when they had to promote and protect the interests of the employer (institution, ministry) rather than those of their patients.

The multivariate analysis for the estimation of indicators associated with ‘Interests of the institution’ can be seen in Table 2.

This model included Age (B = −0.29) and Profession (B = 1.094), completed with the value of Constant (B = −1.597). By interpreting the types of associations, we notice that with the increase of Age by one year, the probability of the respondent reporting the ethical dilemma concerning the interests of the institution where they work decreases by 2.8% (OR = 0.972 95% CI, 0.960, 0.983). Doctors will report cases of non-compliance on the part of the institution almost 3 times more often than nurses (OR = 2.988, 95% CI 2.183, 4.089). It is important to mention the relatively narrow confidence intervals and the fact that the described relationships fit within the logic of the research.

A group of 83 respondents (7.8%, 95% CI, 6.3, 9.5) admitted that they had faced dual loyalty dilemmas when they had to provide assistance to a terminally ill patient or a very sick homeless person. This dilemma arose for some respondents who had doubts about justifying the waste of resources, effort, and time of medical staff on patients who had no chance of survival or for whom the hospital would not be reimbursed because they did not have medical insurance. These dilemmas were mostly motivated by the limited resources of medical institutions in the Republic of Moldova, but also by the acute lack of medical personnel. It becomes obvious that doctors experience situations in which they must justify the equitable distribution of resources, thereby resulting in ethical dilemmas.

The multivariate analysis for the estimation of indicators associated with ‘Unnecessary treatment’ can be seen in Table 3.

The model included Age (B = −0.22) and Profession (B = 1.451) and the field of Pediatrics, completed with the value of Constant (B = −3.968). We observe that doctors will report cases of ‘Unnecessary treatment’ 4.3 times more often than nurses (OR = 4.268, 95% CI, 2.217, 8.216). Respondents from the specialty of Pediatrics reported such cases 4.6 times less often compared with other specialties (OR = 0.218, 95% CI, 0.05, 0.905). The confidence intervals are relatively narrow, and the described relationships fit the logic of the research.

The study identified that the decision-making process of the medical staff is also influenced by abusive interference from their superiors, which influences health care professionals to take certain decisions, especially in favor of the interests of the institution instead of those of the patient. It is alarming that more than half of the respondents (68.4%) indicated that they had faced situations of inappropriate influence from their superiors (e.g., giving preference to some patients, limiting some resources, influencing decisions, etc.), and 21.8% (N = 233, 95% CI, 18.3, 25.7) mentioned that they encountered such situations often or very often.

#### 3.2.2. The Obligation to Promote the Interests of the Insurer

In the Republic of Moldova, the system of Mandatory Insurance for Medical Assistance is implemented, based on the contributions of employees (9% of salary) and state contributions for groups of vulnerable people who are medically insured by the government (children, the elderly, pregnant women, people with disabilities, etc.). The medical insurance fund is managed by the National Medical Insurance Company (CNAM) which annually contracts each medical institution. Medical services in hospitals are paid per case treated, and primary health care is paid per capita. The Republic of Moldova is a country that faces many economic problems. There is a low level of citizen income, high unemployment, and many people are on the verge of poverty. For this reason, payments to the insurance fund are much lower than the real needs of the health system. The prices provided by the CNAM for the services within the contracts with the medical institutions are very low, barely covering the real costs of medical assistance. For this reason, doctors are always asked by the institutional management to save on prescribing treatments and investigations at the expense of the medical institution.

Almost a quarter of the participants (23.3%, N = 249, 95% CI, 20.8, 25.9) admitted that they had to act in the interests of the insurance company, even if they understood that the conditions imposed did not benefit the patient.

The multivariate analysis for the estimation of indicators associated with ‘Interests of the insurance company’ can be seen in Table 4.

The model included Age (B = −0.18) and Profession (B = 1.335), completed with the value of Constant (B = −2.901). We observe that with the increase of Age by one year, the probability of the respondent reporting the ethical dilemma relating to the interests of the insurance company decreases (OR = 0.983 95% CI, 0.970, 0.995). Physicians reported the ethical dilemma of promoting the insurance company’s interests over the patient’s interests almost 3 times more often than nurses (OR = 3.801, 95% CI, 2.651, 5.451). This phenomenon was particularly reported by family doctors and those in the field of oncology.

#### 3.2.3. Protecting a Colleague’s Interests/Guild Loyalty

Dual loyalty can also occur in the context of relationships between medical professionals. Thus, 13.9% (N = 149, 95% CI, 11.9, 16.1) of the participants identified cases when they had to side with a “guild” colleague against the interests of a patient.

If they noticed a medical error or unethical behavior on the part of a colleague, only 14.1% (N = 151, 95% CI, 12.1, 16.3) of the respondents stated that they would report the problem; that is, they would notify their superior about it.

The vast majority (73.4%, N = 785, 95% CI, 70.7, 75.9) indicated that they would prefer to talk to the colleague personally, without notifying the superiors, and 12.5% (N = 134, 95% CI, 10.6, 14.6) of the respondents admitted that they would not get involved, considering that it is not their duty to point out someone else’s mistakes.

#### 3.2.4. Promotion of Personal Financial Interests

The study identified a large number (74.4%, N = 796) of participants who admitted that they faced conflicts of interest in professional relationships, with practically every third respondent (23.9%, N = 256, 95% CI, 20.3, 28.0) rating such cases as frequent or very frequent.

About 18.0% (N = 193, 95% CI, 15.8, 20.4) of the respondents indicated “personal profit” as a characteristic of the climate of the institution where they work, thus emphasizing the predominance of personal financial interests of the respondents.

Sixteen respondents (1.5%, 95% CI, 0.9, 2.4) admitted that they had faced situations of dual loyalty and conflict of interest related to being simultaneously employed in a public and a private institution when they had to promote the private institution for their own financial interest.

Sixty-nine respondents (6.4%, 95% CI, 5.1, 8.0) indicated that certain pharmaceutical companies offered them very attractive and motivating conditions to give preference to prescribing certain medications from certain manufacturers (Table 5).

The model included the Profession (B = 0.229) and the Family Medicine specialty (B = 654), completed with the value of Constant (B = −6.852). We observe that physicians will more frequently report ethical dilemmas related to pharmaceutical companies than nurses (OR = 9.288, 95% CI, 3.389, 25.458). Such dilemmas were most frequently mentioned by family doctors.

### 3.3. The Existence of Procedures for Reporting Problems

Dilemmas and conflicts of interest in professional activity presuppose the need for their correct management within an institution. Once dilemmas are identified, any health care professional should initiate a reporting procedure and seek specialist advice. Our study identified a low rate of health care professionals asking for support when faced with certain ethical dilemmas. Practically half of the respondents (50.6%, N = 541, 95% CI, 47.6, 53.6) preferred to discuss the identified dilemma only with a close colleague, and 40.1% (N = 429, 95% CI, 37.2, 43.1) of the participants declared that they preferred to find solutions on their own without resorting to someone’s help. Relatively few respondents noted that they would seek the help of other specialists, such as the institution’s lawyer (14.1%, N = 151, 95% CI, 12.1, 16.3), the ethics committee (10.8%, N = 116, 95% CI, 9.1, 12.8), or the psychologist (6.3%, N = 67, 95% CI, 4.9, 7.8).

It is alarming that many respondents described the climate of the institution using negative characteristics, such as fear (14.2%, N = 152, 95% CI, 12.2, 16.4) or indifference (22.4%, N = 240, 95% CI, 20.0, 25.0), which indicates employees’ low predisposition toward openness and communication with superiors about the problems and dilemmas they face.

Moreover, 22.1% (N = 236, 95% CI, 19.6, 24.6) of the participants believed that in the institutions where they worked, insufficient and superficial measures were taken to promote the ethical behavior of employees, while 24.7% (N = 264, 95% CI, 22.2, 27.3) were unable to make an assessment with reference to this topic.

## 4. Discussion

Along with the development of contemporary society, there are inherently higher expectations regarding the standard of medical services provided. Political pressure, but also the increasing emergence of patient movements and organizations, promote patient rights to a professional level. Thus, the patient’s complaint and choice have become a priority, influencing the care management policies adopted by institutions, the establishment of transparency, ethical reflection, and legal justification of the actions and decisions taken. As a result, clinicians will increasingly need specific advice on decisions that need to be made in certain cases, both from a legal and a moral perspective [20,21].

### 4.1. The Influence of the Interests of the Medical System

The hospital manager might put the interests of the national health care system and regulatory authorities before the interests of the patient, those of political leaders, employers, or board members, as well as the interests arising from the conditions dictated by the insurance company. Taking into account the fact that a hospital operates depending on the funds at its disposal, there could be situations where the decision to admit a patient or not, as well as the treatments applied, depend on the funds allocated to the institution or the system by which the institution is financed. For example, the most financially “advantaged” patients or patients with better or higher-level insurance may be given preference when the funding system depends on the patients’ ability to pay or on their insurance status [5]. At the same time, if the funding system is dependent on a fixed amount provided per patient or for the care of a population over a certain period of time, patients who require extensive, expensive interventions and treatments might be avoided [5]. A study of doctors in Germany drew attention to the “increasing pressure to consider the economic interests of hospitals when making clinical decisions” [22].

About a quarter of the participants in our study indicated the existence of a conflict between the economic interests of the institution in which they worked and the good of the patient, and about a third of the participants faced ethical dilemmas when they had to ensure the fair access of patients to complex diagnostic investigations and expensive treatments. Doctors were three times more likely to face such dilemmas than nurses, most likely due to the fact that doctors are the ones who recommend medical investigations and treatments to patients. The likelihood of facing these dilemmas decreases with age. This fact can be explained by the traditional paternalistic attitude of older doctors, educated in the Soviet period, when the interests of society were promoted over those of the individual—the patient. As employees of the state, physicians were subject to its discipline and directives; they lacked the control over their own work that is essential to a profession [23]. Even the oath of Soviet doctors speaks of the high responsibility of the physician to “my people and the Soviet Government”, with the diminution of the meaning of the more individualistic, traditional Hippocratic Oath [24].

### 4.2. The Influence of the Interests of the Medical Institution

Another situation, which does not directly concern financial matters, but which emphasizes the priority of the hospital’s interests over the patient’s, is related to the hospital’s reputation. For example, in order to have good quality indicators with a low postoperative 30-day mortality rate, some hospitals in the USA practice prolonged hospitalization and the continuation of treatments that keep the patient alive for more than 30 days postoperatively, without taking into account the patient’s willingness to accept a treatment that unnecessarily prolongs their life [25,26].

The dependence of the institution on the funds and conditions imposed by the insurance company is another situation that may generate ethical dilemmas and dual loyalty in the behavior of doctors. About a quarter of our study participants said they acted in the insurance company’s best interests, with the likelihood decreasing with age. The probability is higher among doctors compared with nurses, with the most vulnerable specialties being family medicine and oncology.

Dual loyalty can also occur when doctors are faced with the dilemma of administering expensive drugs to terminal patients, when their curative potential is questionable, and the hospital will bear high expenses considered unnecessary [27]. In our study, physicians reported cases of dual loyalty by forgoing treatments they considered unnecessary and resource-consuming. Physician respondents mentioned these cases more frequently than nurses, which is a natural result, given that decisions regarding the administration or discontinuation of certain treatments belonged to physicians.

### 4.3. The Influence of Personal Interests

At the opposite end, there are situations where the doctor has concluded a fee-for-service contract with the medical institution. In such cases, there is the temptation to request more procedures for more money, even when the investigations or procedures requested are not in the patient’s best interests [5].

A concrete example of this is the doctor’s involvement in a private analysis or treatment center and directing patients to this center with the aim of supplementing income, and not necessarily because it is in the best interest of the patient. Instead, this results in a financial gain for the doctor and a potential conflict of interest [15].

Similarly, financial considerations may lead some doctors to approach patients differently when working in both public and private practice, with a tendency to promote or refer patients to private practice for financial gain, a situation reported by a small number of participants in our study.

The practices of pharmaceutical companies to offer doctors bonuses for prescribing certain promoted drugs involves the risk of a conflict of interest [5]. In our study, we found that 6.4% of the participants faced dilemmas regarding the prescription of certain drugs at the request of pharmaceutical companies. The specialty with the highest probability of such dilemmas was family medicine, possibly because these specialists have much more freedom in prescribing than doctors in hospitals, where drugs are procured centrally.

Drug companies or those producing medical consumables and devices may have a commercial rather than social interest when funding clinical trials [12]. Medical representatives frequently offer doctors various gifts or finance the publication of articles or their participation in conferences [12]. Although some claim that such practices do not influence the prescription of the drugs provided by the funding companies, others wonder what the purpose would be for pharmaceutical companies to spend enormous sums on such funding as well as paying a commission to medical representatives based on the number of prescriptions prescribed by the doctors they visited [1]. Drug companies often provide doctors with free drug samples to give to patients who cannot afford them. At first glance, this practice might be considered honorable, but since these samples are for new and expensive drugs, what happens when the samples run out and the patient will have to pay more to continue treatment with the new drug rather than following treatment with an equally effective and less expensive generic drug? [1].

### 4.4. Institutional Tools for Addressing Ethical Issues

The need to support doctors in making decisions in situations of dual loyalty, ethical dilemmas, and conflicts of interest was determined at the end of the last century. In a study of UK hospitals, 59% of respondents indicated that they would prefer this advice to be provided by an ethics committee, while 36% stated they would prefer to seek advice from a qualified clinical ethicist. [28]

Initially, there was a lack of opportunity in medical institutions to systematically deal with moral dilemmas associated with medical activity. Gradually, specific methods of ethical analysis were developed to help employees deal with these issues in a more reflective way, through constructive dialogue. Aulisio et al., for example, indicate that ethics consultation helps to identify and analyze the nature of the value conflict or uncertainty and create consensus among the parties involved. The importance of moral reflection on each dilemma or ethical uncertainty is emphasized—not only to develop the professional capacities of the employees, but also to highlight the institutional or organizational problems that lead to the appearance or accentuation of ethical problems [29].

Even if the objectives of the ethical consulting structures in a medical institution seem to be clear, it remains to be determined to what extent these objectives are understood and accepted by key personnel in the institution. Some empirical studies highlight the objectives considered important in practice. For example, the findings of a survey conducted in more than 600 US hospitals show that the following are considered important goals of ethics consultation: protecting patients’ rights (94%), resolving actual or imminent conflicts (77%), actions to improve the quality of care (75%), and increasing patient and family satisfaction (68%) [30].

Dauwerse et al. (2013) proposed four objectives of ethical consultation in hospitals: (1) Encouraging an ethical climate; (2) Promoting an accountable and transparent organization; (3) Increasing professional awareness of the ethical dimension of health care; and (4) Providing good quality assistance [17].

The promotion of ethical values in the institution must be a continuous process and must be monitored by those responsible for the institution’s ethics. Through systematic democratic discussions, employees should be encouraged to talk about the ethical dilemmas they encounter in daily practice. For this, channels and structures must be created to help health workers deal with the moral dilemmas they may encounter and, at the same time, benefit from an increase in job satisfaction. At the same time, the medical institution will achieve better results in terms of patient satisfaction and employee retention [31].

Improving the quality of ethics within medical institutions is becoming a novel process and trend at the international level, which means that the practices in medical institutions are consistent with declared and accepted ethical standards and norms, as well as with the expectations of the institution and the medical team [32].

In a responsible organization, institutional policies must be promoted that also include ethical aspects, especially those that would help managers of a medical institution with decision making when faced with ethical dilemmas. Such dilemmas could include the distribution of funds, types of services provided to certain vulnerable groups of patients, patient selection, etc. Such choices should be based on fair reasoning and dialogue, so that the manager’s decision is made after consultation with employees invited to express their own points of view. Thus, the need for institutional policies regarding the way and procedure to approach ethical issues within the organization, at all levels, becomes evident [18].

Through ethical consultations, practitioners become increasingly aware and better able to recognize ethical aspects in daily practice; they also become aware of the fact that ethical choices are sometimes made without deeper consideration of such choices. Ethical reflection promotes an approach to a situation, for example, by asking oneself: Was I fair enough in my decision? Through ethical consultation, ethical reflection is stimulated, which will directly influence the development of professionalism in a positive way [30].

Our study determined that sometimes doctors ignore the importance of their own ethical reflection and prefer to execute what has already been decided and documented by others. Consequently, self-responsibility is frequently minimized due to the belief that *I personally cannot influence things at the organizational level*. Thus, for the administrators of a medical institution, it is important to promote a conducive context that would stimulate the confidence of the staff as well as promoting transparency and ethical dialogue in decision making. This type of communication must be promoted as a continuous, contextual, and situational process, that seeks to answer questions about “what is right” in daily clinical activity. It is imperative that medical institutions work on support for ethics consultation, which can yield multiple benefits, from developing an ethical climate and fostering professionalism to promoting an accountable organization. Ideally, the choice of such goals is the product of discussions between managers and practitioners within the health care institution, including ethicists. In this way, commitment to ethical consulting can be stimulated [17,33].

### 4.5. Strengths and Limitations of the Study

This is the first study of this type carried out in the Republic of Moldova. The study was conducted on a large group of participants (doctors and nurses) from all medical institutions in this country. However, the study has a number of limitations that we present below.

Completing the online questionnaire did not allow us to keep the representativeness of the participants in the study according to the ratio existing at the country level between specialties, the type of institution, as well as between the proportion of personnel with secondary and higher education. Thus, among the respondents, we determined an increased rate of doctors (almost 2/3 of the analyzed sample) and a reduced rate of nurses (almost 1/3 of the analyzed sample), which contradicts the distribution of these categories in the target population, where the number of nurses is 2 times higher than the number of doctors. However, these facts may signal the increased sensitivity of doctors to the problem addressed in this study, with the latter having more responsibilities toward patients and the administration.

Another limitation may also be the probability of the participants’ formal answer, as they may not be sufficiently honest. Lastly, we appreciate that the models developed by us can be completed and require validation in further studies.

## 5. Conclusions

Ethical health care is a continuous contextual process, based on concrete experiences and relationships with different stakeholders (e.g., patients, relatives, managers, society, donors, development partners, etc.). Employees of medical institutions are, and always will be, faced with ethical problems and conflicts in their daily work, even if sometimes they are not aware of the moral dimension of their decisions and behavior. The persistence of conflicts of interest, improper influence, uncertainties, and ethical dilemmas in the decision-making process in the practice of medical workers significantly influences their moral status. This creates an internal conflict of values and contradictions, which can affect the behavior and integrity of employees as well as the reputation of the medical institution.

The results of our study show that in the medical institutions in the Republic of Moldova there are significant gaps in the organization of the ethical dimension of the institutions where the study participants work, namely, the presence of ethical dilemmas, dual loyalty, and conflicts of interest, the lack of necessary support for ethical decision-making processes, and an unfavorable institutional climate. The need for serious measures to promote an institutional climate based on trust, openness to discussion, and constructive dialogue becomes evident.

Thus, managers should adopt institutional policies and clear actions for the implementation of ethical tools to identify, manage, and prevent such situations. The promotion of institutional policies to deal with moral dilemmas, the provision of assistance, and ongoing consultation support for medical workers in the process of making difficult moral decisions must become a priority for current health managers. In the process of organizing the activity of medical institutions, the need to establish and promote a separate field dedicated to these needs is highlighted, namely, that of institutional ethics management.

## Figures and Tables

**Table 1:** Age of participants.

Age, years		**Physicians**	**Nurses**	**Non-Medical Staff**
	Minimum	24	21	23
	Maximum	73	68	68
	Mean	46	44	45
	Standard deviation	12	11	11
	Median	44	45	43

**Table 2:** Estimation of indicators associated with ‘Interests of the institution’. Multivariate analysis.

	B	S.E.	Wald	df	Sig.	Exp(B)	95% CI for Exp(B)	
							Lower	Upper
Age, years	−0.029	0.006	21.401	1	0.000	0.972	0.960	0.983
Medical Profession	1.094	0.160	46.754	1	0.000	2.988	2.183	4.089
Constant	−1.597	0.379	17.772	1	0.000	0.202		

**Table 3:** Estimation of indicators associated with ‘Unnecessary treatment’. Multivariate analysis.

	B	S.E.	Wald	df	Sig.	Exp(B)	95% CI for Exp(B)	
							Lower	Upper
Age, years	−0.022	0.010	4.716	1	0.030	0.978	0.959	0.998
Profession	1.451	0.334	18.849	1	0.000	4.268	2.217	8.216
Pediatrics	−1.522	0.726	4.396	1	0.036	0.218	0.053	0.905
Constant	−3.968	0.749	28.085	1	0.000	0.019		

**Table 4:** Estimation of indicators associated with ‘Interests of the insurance company’. Multivariate analysis.

	B	S.E.	Wald	df	Sig.	Exp(B)	95% CI for Exp(B)	
							Lower	Upper
Age, years	−0.018	0.007	7.122	1	0.008	0.983	0.970	0.995
Profession	1.335	0.184	52.706	1	0.000	3.801	2.651	5.451
Family Medicine	0.817	0.190	18.441	1	0.000	2.264	1.559	3.287
Oncology	0.955	0.302	9.972	1	0.002	2.599	1.437	4.700
Constant	−2.901	0.430	45.502	1	0.000	0.055		

**Table 5:** Estimation of the indicators associated with ‘The interests of the pharmaceutical company’. Multivariate analysis.

	B	S.E.	Wald	df	Sig.	Exp(B)	95% CI for Exp(B)	
							Lower	Upper
Profession	0.229	514	18.768	1	0.000	9.288	3.389	25.458
Family medicine	654	293	4.973	1	0.026	1.924	1.082	3.418
Constant	6.852	0.010	46.024	1	0.000	0.001		

## Data Availability

Data are available, on request, from the corresponding author.
